# Evaluation of Alveolar Bone Loss With ECR Using Cone‐Beam Computed Tomography: A Cross‐Sectional Study

**DOI:** 10.1155/ijod/5186337

**Published:** 2026-09-12

**Authors:** Ana Paula de Medeiros Silva, Thalles Gabriel Germano Lima, Ana Beatriz Leitão de Albuquerque Wanderley, Monikelly do Carmo Chagas Nascimento, Daniela Siqueira Lopes

**Affiliations:** ^1^ Department of Dentistry, Pernambuco School of Dentistry, University of Pernambuco (FOP/UPE), Recife, Pernambuco, Brazil, ufpe.br; ^2^ Division of Oral Radiology, São Leopoldo Mandic Research Institute, Campinas, São Paulo, Brazil

**Keywords:** alveolar bone loss, cone-beam computed tomography, diagnosis, root resorption

## Abstract

Alveolar bone loss (ABL) and external cervical resorption (ECR) are destructive processes that can coexist and compromise periodontal prognosis. Cone‐beam computed tomography (CBCT), enables assessment of resorption severity and bone loss patterns, overcoming the limitations of two‐dimensional radiography. This study aimed to evaluate ABL and its correlation with ECR using CBCT. This analytical, cross‐sectional, observational study used images to database spanning January 2019 to April 2025, acquired with a CBCT scanner. A total of 9208 examinations were screened. After applying the inclusion criteria, 64 patients were enrolled, comprising 75 teeth diagnosed with ECR. Sociodemographic and clinical data, ECR characteristics, and ABL patterns and classification were analyzed. The findings demonstrated a statistically significant correlation between ABL and ECR according to tooth groups, with stronger associations observed in premolars and molars (10.7%), subcrestal lesion height (41.3%), and circumferential distribution 90° to 180° (44.0%). ABL was associated with age group and lesion circumferential distribution (*p*  < 0.05). The horizontal pattern of bone loss was the most prevalent (54.7%), with significant associations between severity and age group (*p* = 0.022) and lesion height (*p* = 0.007). An association was also observed between lesion height and periodontal condition (*p* = 0.043). In conclusion, ECR performed a significant correlation with ABL in the studied population (*p*  < 0.05). Three‐dimensional characterization of ECR lesions and their relationship with bone loss patterns and periodontal status underscores the critical role of imaging in the early detection of potentially progressive lesions.

## 1. Introduction

Dysbiotic bacterial communities and the host immune response, which are frequently associated with poor oral hygiene in clinical practice, contribute to a chronic inflammatory process that results in a reduction of the alveolar crest and subsequent alveolar bone loss (ABL). The severe condition is also the most prevalent among populations and is classified by pattern as horizontal or vertical based on the number of remaining bony walls [1, 2].

In addition, another clinical disorder is the external cervical resorption (ECR), which may lead to progressive tooth destruction and affect the patient’s quality of life. Usually begins in the cervical third of the root and extends toward the root canal as well as vertically toward the root structure [3]. Damage to the periodontal ligament and subepithelial cementum is rarely associated, thereby activating clastic cells and initiating a resorptive cascade [4, 5].

The resorptive process is dynamic and may involve the periodontal tissues, potentially altering alveolar bone. However, diagnosis remains challenging and may be influenced by periodontal conditions [4]. Early‐stage or interproximal lesions are often difficult to detect, whereas cavitated cervical defects on the buccal and lingual surfaces are more readily identified by direct visualization or periodontal probing [6].

Imaging systems provide valuable complementary support to clinical decision‐making and treatment. However, the assessment of ECR using two‐dimensional imaging is limited by geometric distortion, anatomical superimposition, and insufficient characterization of dental anatomy [7]. Cone‐beam computed tomography (CBCT) provides three‐dimensional (3D) visualization, reducing distortion and anatomical overlap, thereby enhancing diagnostic accuracy and improving treatment planning [8].

CBCT, such as PreXion 3D (PreXion Corporation, Japan), improves the assessment of probing depth, defect pattern (vertical and/or horizontal), classification of ABL, and the detection of ECR [1, 2, 9]. The specificity of CBCT depends on acquisition parameters, such as exposure settings, voxel configuration, detector sensitivity, and tomographic reconstruction algorithms, as well as on examiner experience [3]. Features include continuous X‐ray emission and an integrated motion‐artifact system, enhancing image quality and diagnostic reliability [8, 10].

Distance between the cementoenamel junction (CEJ) and the alveolar bone crest, as observed in postorthodontic bone dehiscences and periodontitis, is associated with a morphological pattern characterized by cervical resorptive processes, suggesting an overlap of etiological factors and clinical manifestations [11]. However, a scarcity of studies correlating ABL and ECR within a single three‐dimensional approach limits the understanding of the relationship between bone destruction and cervical resorption and the identification of ABL patterns as potential predictive markers of ECR.

The present study aimed to evaluate ABL and its correlation with ECR using CBCT images. The hypothesis tested was that ECR severity is associated with the presence of adjacent ABL.

## 2. Materials and Methods

### 2.1. Study Design and Ethical Approval

This analytical cross‐sectional observational study was conducted in accordance with the Declaration of Helsinki and approved by the Research Ethics Committee of the University of Pernambuco (approval number 7.129.157), in accordance with Resolution No. 466/2012 of the National Health Council/Ministry of Health. The study was reported following the STROBE guidelines for observational studies [12].

### 2.2. Sample Selection

A total of 9208 CBCT image records, obtained between January 2019 and April 2025 using the PreXion 3D system (PreXion Corporation, Japan), under the following operating parameters: 90 kVp, 4 mA, exposure time of 33.5 s, voxel size of 0.100 mm^3^, and field of view (FOV) of 50 mm × 50 mm. Images were analyzed using PreXion 3D Viewer software (PreXion Corporation, Japão. Patient identification codes (IDs), examination dates, and sociodemographic variables (age, sex, dental group, affected region, reason for examination request, and referring professional specialty) were recorded in a Microsoft Excel spreadsheet.

**Figure 1:**
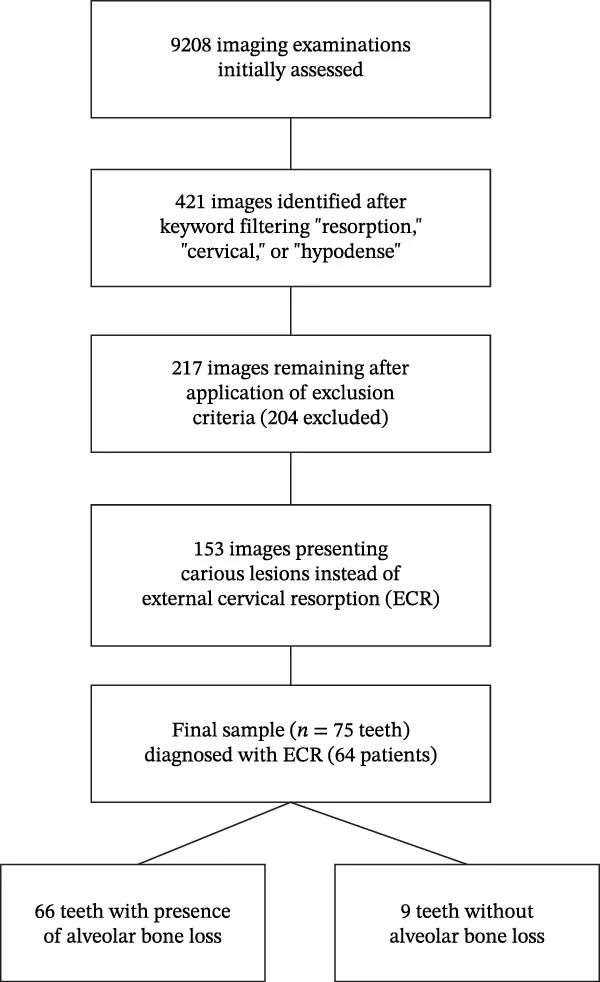
Study phases of sample selection.

To identify cases of ECR, digital radiology reports were retrospectively searched using the terms “resorption,” “cervical,” and “hypodense.” Subsequently, all CBCT images were screened for ABL. This process initially yielded 421 tomographic reports containing all three search terms. After removing duplicates and applying eligibility criteria, 217 cases were retained. Further DICOM file evaluation led to the exclusion of 153 cases due to caries‐related features, resulting in a final sample of 64 patients and 75 ECR‐affected teeth (Figure 1).

#### 2.2.1. Inclusion Criteria

CBCT image records:

• Adult patients of both genders presenting one or more permanent teeth with ECR, regardless of the presence or absence of ABL.
• Teeth with a clearly identifiable CEJ on at least one tooth surface in the three‐dimensional CBCT images.
• Permanent teeth fully developed.

#### 2.2.2. Exclusion Criteria

CBCT image records:

• Artifacts and/or lesions in the region of interest impair the evaluation of the structures.
• Impacted or partially erupted teeth.
• Teeth located at the periphery of the FOV and with extensive restorations.

#### 2.2.3. Examiner Training and Calibration

Three calibrated examiners: an endodontist, a periodontist, and an experienced radiologist, carried out the assessments. A representative subset corresponding to 10% of the sample was evaluated [3, 12]. Inter‐ and intra‐examiner agreement was assessed using the Kappa statistic, with a significance level set at 5% (*α* = 0.05) [6]. Reliability was additionally estimated using Krippendorff’s alpha [3], yielding values of 0.907 in the first assessment and 0.815 in the second, indicating almost perfect agreement. Following calibration, all CBCTs included in the study were assessed by one calibrated examiner.

### 2.3. ECR Analysis

Hypodense images were observed in the three orthogonal planes, originating from the cervical third of the root, and were analyzed [7]. Cases presenting hypodense or hyperdense images with reparative mineralized tissue, extending to the middle and apical thirds and circumferentially involving the root canal, were included [9]. Lesions were differentiated from dental caries by excluding rounded, focal hypodense areas with a mushroom‐shaped appearance, characterized by a narrow pathway through enamel and a broader area of demineralized dentin [13].

**Figure 2:**
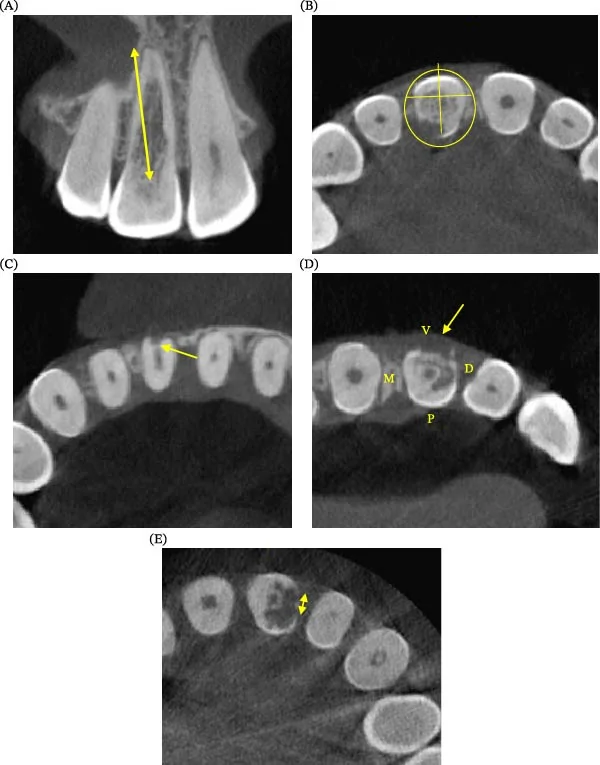
Measurements of external cervical resorption (ECR): coronal section—(A) lesion height; axial section—(B) circumferential distribution, (C) proximity to the root canal, (D) location of the entry portal, and (E) size of the entry portal.

Teeth were classified according to the Patel classification, allowing assessment of lesion height, circumferential distribution, and relationship with the pulp [14, 15]. The apicocoronal extent of the lesion relative to the alveolar crest was categorized as supracrestal or subcrestal. The circumferential extent was classified as A (<90°), B (90°–180°), C (180°–270°), or D (>270°). The anatomical involvement was categorized as confined to dentin (D) or associated with pulpal involvement (P). In addition, the number of entry portals, their location (buccal, lingual, palatal, mesial, or distal), and the size of each entry portal (≤1 or >1 mm) were assessed (Figure 2).

### 2.4. ABL Analysis

ABL was assessed by measuring the distance between the cemento‐enamel junction (CEJ) and the alveolar bone crest using the PreXion 3D Viewer software (PreXion Corporation, Japan). Mesial and distal surfaces and buccal and lingual surfaces were evaluated in the cross‐sectional view [16]. The most coronal level at which the periodontal ligament space width remained within normal limits (<2 mm) was the endpoint [17].

**Figure 3:**
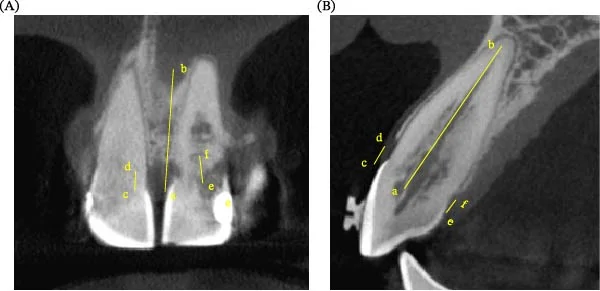
Measurements of cementoenamel junction (CEJ) to alveolar crest to determine alveolar bone loss (ABL). (A) Coronal section: a–b, CEJ to root apex; d–c, CEJ to mesial alveolar crest; e–f, CEJ to distal alveolar crest. (B) Sagittal section: a–b, CEJ to root apex; d–c, CEJ to buccal alveolar crest; e–f, CEJ to palatal alveolar crest.

ABL was assessed in coronal sections and categorized by pattern (horizontal or vertical) and extent of root involvement as slight (≤15%), moderate (16%–30%), or severe (>30%) [1, 2]. ECR was considered associated with ABL when both defects involved the same root surface. This classification further required evidence of communication between the ECR and the periodontal space, indicated by thickening of the periodontal ligament observed across all tomographic sections (Figure 3).

### 2.5. Statistical Analysis

Categorical variables are presented as frequencies and percentages, whereas continuous variables are expressed as mean ± standard deviation. Associations between ECR and ABL variables were examined using nonparametric tests (Mann–Whitney and Kruskal–Wallis) or categorical tests (Pearson’s chi‐square or Fisher’s exact test), according to the distribution and nature of the variables. Statistical significance was established at *p*  < 0.05.

To assess factors associated with ABL directly related to ECR, a linear mixed‐effects model with a binomial distribution and logit link function was executed. Age group, sex, tooth group, lesion height, circumferential lesion distribution, and lesion proximity to the root canal were included as fixed effects. As the 75 teeth evaluated belonged to 64 patients, patient was included as a random effect, with a random intercept to account for the potential correlation between teeth from the same patient. Associations were expressed as adjusted odds ratios (ORs) with corresponding 95% confidence intervals (95% CIs).

## 3. Results

### 3.1. Characteristics of the Study Participants

In the analyzed subpopulation, ECR was observed in patients with a mean age of 46.1 years (±17.6), with a higher prevalence between 40 and 50 years, followed by 60 years or older (*p* ≤ 0.05). The sample was similar between genders, with 53.1% males and 46.9% females (*p* ≥ 0.05). The maxilla was the most frequently involved anatomical region. Maxillary incisors were the most affected teeth (90%), followed by maxillary canines (62.5%) and maxillary molars (52.3%). These findings were included in the association analysis with the other variables (*p* ≤ 0.05), as shown in Table 1.

**Table 1:** Characteristics of patients, analyzed region, and tooth group.

Variable	*n* (%)	*p*‐Value
Total	64 (100.0)	—
Results by patient		
Age group	—	*p* ^a^ = 0.024 ^∗^
Up to 29	13 (20.3)	—
30 or older	51 (79.7)	—
Total	64 (100.0)	—
Gender	—	*p* ^a^ = 0.133
Male	34 (53.1)	—
Female	30 (46.9)	—
Total	64 (100.0)	—
By anatomical region analyzed		
Results		
Anatomical region requested	—	*p* ^a^ < 0.001 ^∗^
Maxilla	49 (65.3)	—
Mandible	19 (25.3)	—
Both	7 (9.3)	—
Total	75 (100.0)	—
Tooth group	—	*p* ^a^ = 0.004 ^∗^
Incisors	30 (40.0)	—
Canines	16 (21.3)	—
Premolars	8 (10.7)	—
Molars	21 (28.0)	—
Total	75 (100.0)	—

^a^Chi‐square test for comparison of proportions in a single sample.

^∗^Significant difference at the 5.0% level.

**Figure 4:**
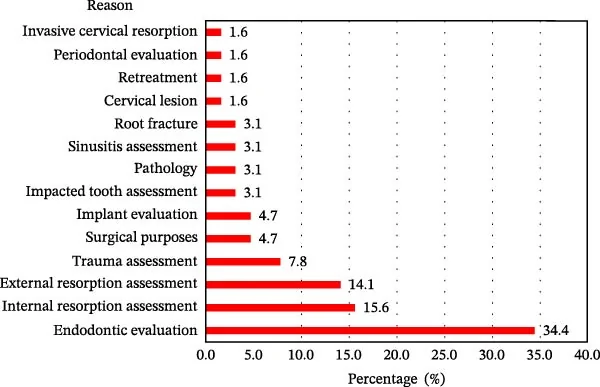
Frequency of the reason for referral.

**Figure 5:**
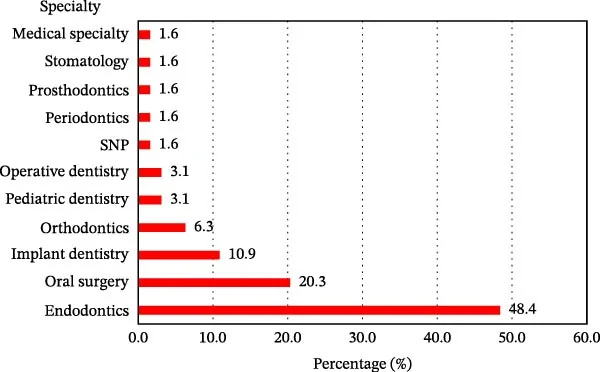
Percentage frequency of the specialty.

Endodontic evaluation accounted for the highest proportion of referrals for tomographic examinations in which ECR lesions were identified. However, ECR was not the primary diagnostic suspicion in all cases. Medical specialties, Stomatology, and Prosthodontics accounted for lower frequencies (1.6%, respectively) (Figures 4 and 5).

### 3.2. Characteristics and Classification of ECR

Regarding lesion height, subcrestal involvement accounted for the highest frequency (41.3%), whereas the lowest frequency was observed when the lesion extended to the apical third (6.7%). Regarding circumferential distribution, the greatest involvement occurred between 90° and 180°. Furthermore, concerning the proximity of the lesion to the root canal, dentin‐confined involvement was the most frequent finding, accounting for 64% of cases (*p* ≤ 0.05).

**Figure 6:**
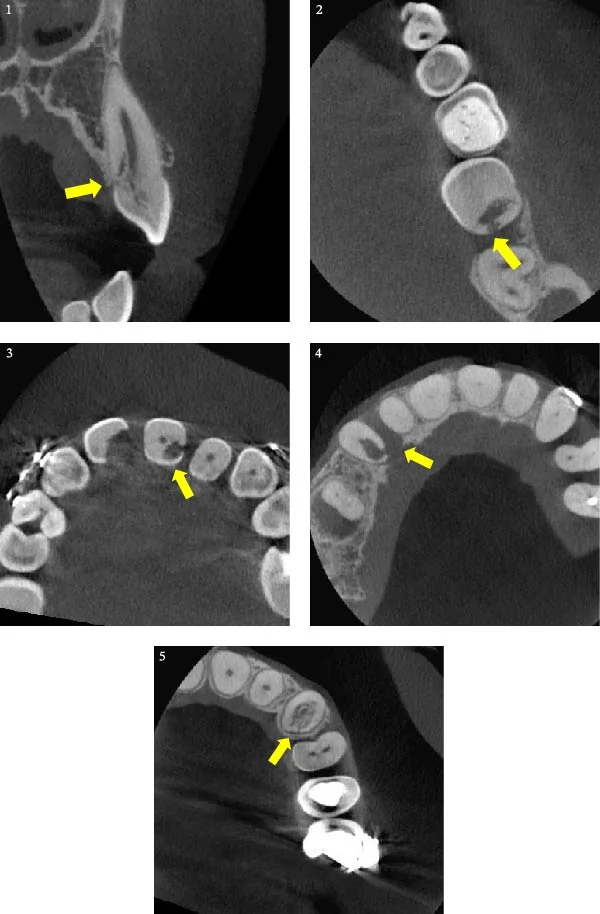
Representative images of features associated with external cervical resorption (ECR). Arrows indicate (1–5): (1) subcrestal lesion level; (2) circumferential distribution ranging from 90° to 180°; (3) lesion confined to dentin; (4) entry portal located on the palatal surface; and (5) entry portal measuring <1 mm.

**Figure 7:**
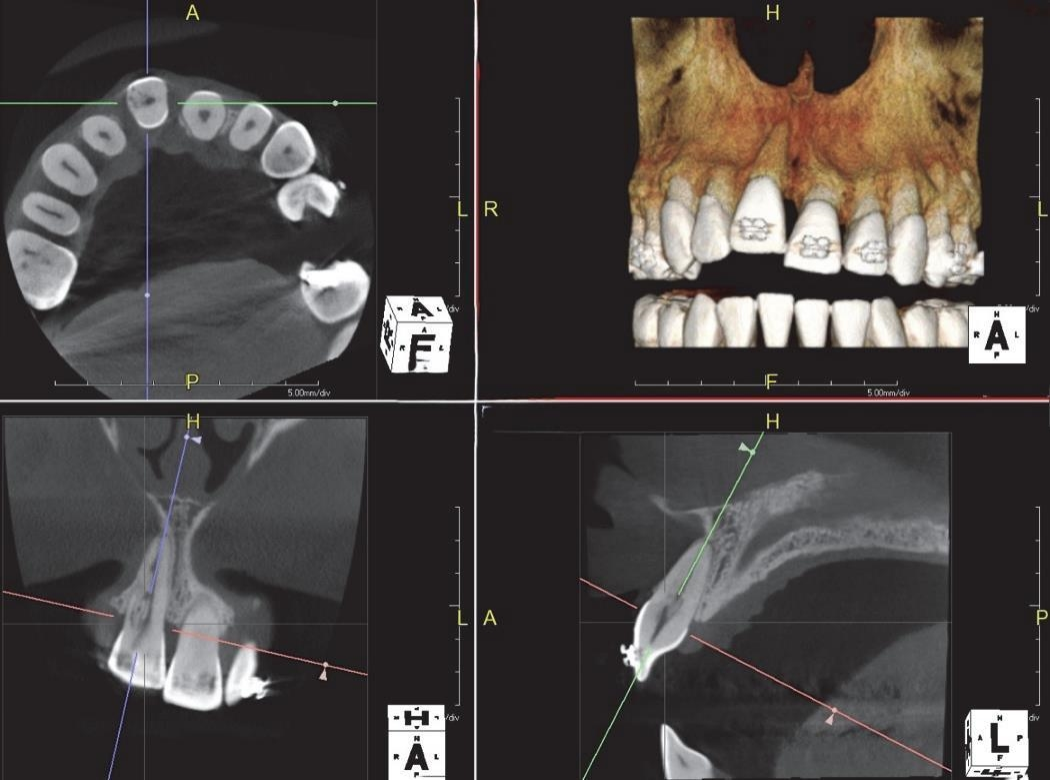
Image illustrating category 2Bd of the Patel classification, as one of the findings of the study.

The most frequent location of the entry portal was the palatal/lingual surface (36%), measuring less than 1 mm. Other locations had lower proportions, without significant differences among them (Table 2 and Figure 6). According to Table 3, type 2Bd was the most prevalent Patel classification among teeth with ECR (14.7%), followed by type 2Ad (12%), with observed differences being statistically significant (*p* ≤ 0.05) (Figure 7).

**Table 2:** Assessment of ECR characteristics.

Variable	*n* (%)	*p*‐Value
Total	75 (100.0)	—
Lesion height	—	*p* ^a^ < 0.001 ^∗^
Supracrestal	21 (28.0)	—
Subcrestal	31 (41.3)	—
Extending to the middle third	18 (24.0)	—
Extending to the apical third	5 (6.7)	—
Circumferential distribution of the lesion	—	*p* ^a^ < 0.001 ^∗^
<90	19 (25.3)	—
90–180	33 (44.0)	—
180–270	8 (10.7)	—
>270	15 (20.0)	—
Proximity of the lesion to the root canal	—	*p* ^a)^ = 0.015 ^∗^
Within dentin	48 (64.0)	—
With pulpal involvement	27 (36.0)	—
Location of the portal of entry on the surface	—	—
Bucal	—	*p* ^a^ < 0.001 ^∗^
Yes	19 (25.3)	—
No	56 (74.7)	—
Palatal/lingual	—	*p* ^a^ = 0.015 ^∗^
Yes	27 (36.0)	—
No	48 (64.0)	—
Mesial	—	*p* ^a^ < 0.001 ^∗^
Yes	19 (25.3)	—
No	56 (74.7)	—
Distal	—	*p* ^a^ = 0.004 ^∗^
Yes	25 (33.3)	—
No	50 (66.7)	—
Lesion size at the portal of entry	—	—
Bucal	—	*p* ^a^ = 0.008 ^∗^
<1 mm	12 (63.2)	—
>1 mm	6 (31.6)	—
No reported	1 (5.3)	—
Total	19 (100.0)	—
Palatal/lingual	—	*p* ^a^ = 0.564
<1 mm	15 (55.6)	—
>1 mm	12 (44.4)	—
Total	27 (100.0)	—
Mesial	—	*p* ^a^ = 0.004 ^∗^
<1 mm	13 (68.4)	—
>1 mm	4 (21.1)	—
No reported	2 (10.5)	—
Total	19 (100.0)	—
Distal	—	*p* ^a^ = 0.072
<1 mm	17 (68.0)	—
>1 mm	8 (32.0)	—
Total	25 (100.0)	—

^a^Chi‐square test for the comparison of proportions in a single sample.

^∗^Significant difference at the 5.0% level.

**Table 3:** Distribution of ECR classification.

Variable	*n* (%)	*p*‐Value
Total	75 (100.0)	—
Patel classification	—	*p* ^a^ < 0.001 ^∗^
1BD	8 (10.7)	—
1BP	4 (5.3)	—
1CD	1 (1.3)	—
1DP	1 (1.3)	—
2AD	9 (12)	—
2AP	1 (1.3)	—
2BD	11 (14.7)	—
2BP	6 (8.0)	—
2CD	2 (2.7)	—
2DP	2 (2.7)	—
3AD	2 (2.7)	—
3BD	2 (2.7)	—
3BP	2 (2.7)	—
3CD	3 (4.0)	—
3CP	1 (1.3)	—
3DP	8 (10.7)	—
4CD	1 (1.3)	—
4DP	2 (2.7)	—

^a^Chi‐square test for comparison of proportions in a single sample.

^∗^Significant difference at the 5.0% level.

### 3.3. ABL Assessment

**Figure 8:**
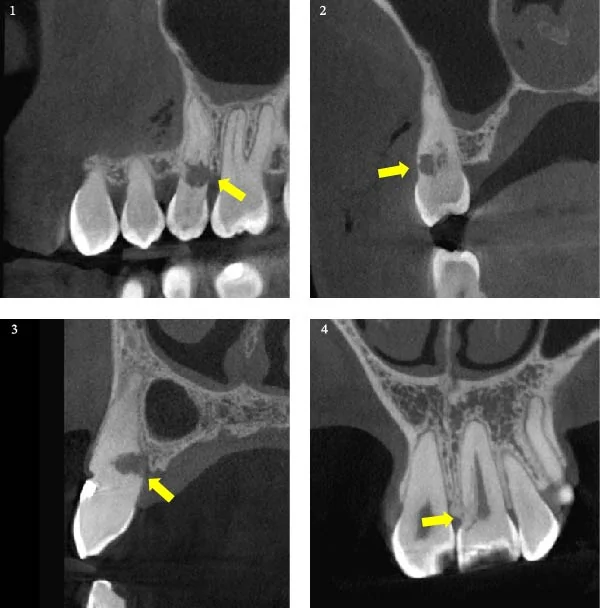
Illustrative image where the arrows indicate, from 1 to 4: (1) horizontal pattern of bone loss; (2) bone loss classified as severe; (3) bone loss directly associated with external cervical resorption; and (4) communication between the resorptive area and the periodontal space.

In the evaluation of ABL in teeth with ECR, a predominance of the horizontal pattern (54.7%) and of cases classified as severe (60%) was observed. A higher prevalence was also noted in findings where bone loss was directly associated with ECR, as well as a hightest periodontal space communication (*p* ≤ 0.05) (Table 4 and Figure 8).

**Table 4:** Assessment of alveolar bone loss (ABL) data of the analyzed teeth.

Variable	*n* (%)	*p*‐Value
Total	75 (100.0)	—
Pattern of bone loss	—	*p* ^a^ < 0.001 ^∗^
Vertical	25 (33.3)	—
Horizontal	41 (54.7)	—
No loss	9 (12.0)	—
Bone loss classification	—	*p* ^a^ < 0.001 ^∗^
Mild	12 (16.0)	—
Moderate	18 (24.0)	—
Severe	45 (60.0)	—
Bone loss directly related to external cervical resorption	—	*p* ^a^ < 0.001 ^∗^
Yes	59 (78.7)	—
Not	16 (21.3)	—
Communication of the resorption with the periodontal space	—	*p* ^a^ < 0.001 ^∗^
Yes	52 (69.3)	—
Not	23 (30.7)	—

^a^Chi‐square test for the comparison of proportions in a single sample.

^∗^Significant difference at the 5.0% level.

### 3.4. ABL and ECR Correlation

A significant association between ABL directly related to ECR and lesion morphology (height and circumferential distribution) (Fisher’s exact test showed *p*  < 0.05). Pearson’s chi‐square test showed no significant association with lesion proximity to the root canal (*p*  > 0.05) (Table 5).

**Table 5:** Assessment of the occurrence of ABL directly related to ECR according to age group, gender, tooth group, lesion height, circumferential distribution, and proximity of the lesion to the root canal.

Variable	Alveolar bone loss directly associated with external cervical resorption (ECR)		Total	*p*‐Value	OR (95% CI)
	Yes	No			
	*n* (%)	*n* (%)	*n* (%)		
Overall group	59 (78.7)	16 (21.3)	75 (100.0)	—	—
Age group	—	—	*—*	*p* ^a^ = 0.013 ^∗^	—
Up to 29 years	8 (53.3)	7 (46.7)	15 (100.0)	—	(1.0)
30 years or older	51 (85.0)	9 (15.0)	60 (100.0)	—	5.0 (1.4–17.1)
Gender	—	—	—	*p* ^b^ = 0.825	—
Male	35 (79.5)	9 (20.5)	44 (100.0)	—	1.1 (0.4–3.5)
Female	24 (77.4)	7 (22.6)	31 (100.0)	—	1.00
Tooth group	—	—	—	*p* ^a^ = 0.015 ^∗^	—
Incisors/canines	32 (69.6)	14 (30.4)	46 (100.0)	—	1.0
Premolars/molars	27 (93.1)	2 (6.9)	29 (100.0)	—	5.9 (1.2–28.3)
Lesion height in relation to crown/root	—	—	—	*p* ^a^ = 0.043 ^∗^	—
Supracrestal	18 (85.7)	3 (14.3)	21 (100.0)	—	3.9 (0.9–17.0)
Subcrestral	27 (87.1)	4 (12.9)	31 (100.0)	—	4.3 (1.1–16.6)
Extending to the apical third	14 (60.9)	9 (39.1)	23 (100.0)	—	1.0
Circumferential distribution of the lesion	—	—	—	*p* ^a^ = 0.012 ^∗^	—
Up to 180	45 (86.5)	7 (13.5)	52 (100.0)	—	4.1 (1.3–13.1)
>180	14 (60.9)	9 (39.1)	23 (100.0)	—	1.0
Proximity of the lesion to the root canal	—	—	—	*p* ^b^ = 0.888	—
Confined to dentin	38 (79.2)	10 (20.8)	48 (100.0)	—	1.1 (0.3–3.4)
With pulpal involvement	21 (77.8)	6 (22.2)	27 (100.0)	—	1.0

^a^Fisher’s exact test.

^b^Pearson’s chi‐square test.

^∗^Significant difference at the 5.0% level.

Subcrestal lesions were associated with a higher bias of ABL (OR = 4.3) compared with lesions extending to the apical third. Similarly, ECR lesions with circumferential involvement of 90° to 180° showed a higher odds of ABL (OR = 4.1) than those with involvement >270°. However, no significant association was observed between lesion proximity to the root canal and ABL (*p*  > 0.05) (Table 5).

**Figure 9:**
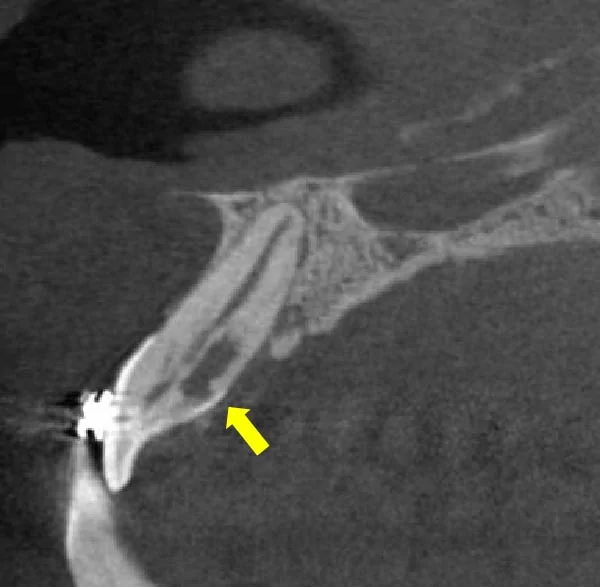
Illustrative image of the relationship between bone loss and external cervical resorption at the subcrestal level.

**Figure 10:**
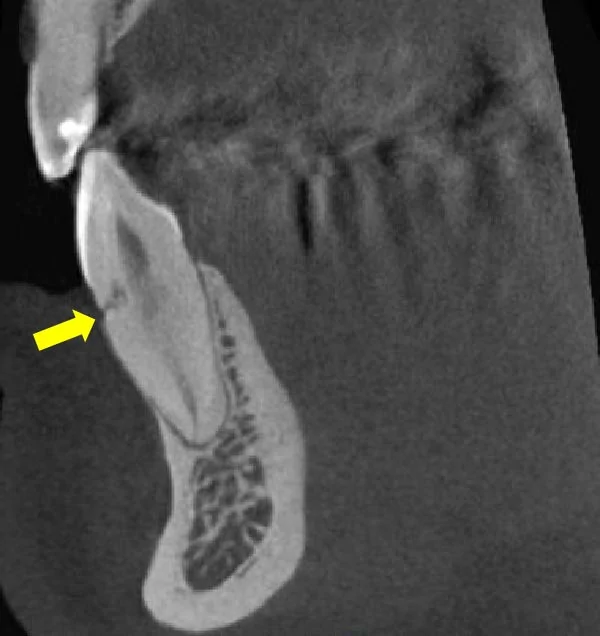
Illustrative image of the relationship between severe bone loss and subcrestal lesion height.

Findings concern ABL and ECR. The Kruskal–Wallis test showed no significant association with the ABL pattern (*p*  > 0.05), but a significant association with lesion height (*p*  < 0.05). No significant association was observed between circumferential distribution and ABL (*p*  > 0.05). The Mann–Whitney *U* test also showed no significant association between lesion next root canal and ABL (*p*  > 0.05) (Tables 6 and 7 and Figures 9 and 10).

**Table 6:** Evaluation of the ABL pattern according to age group, gender, tooth group, lesion height, circumferential distribution, and proximity to the root canal.

Variable	Pattern of bone loss			Total	*p*‐Value
	Vertical	Horizontal	Bone loss		
	*n* (%)	*n* (%)	*n* (%)	*n* (%)	
Total group	25 (33.3)	41 (54.7)	9 (12.0)	75 (100.0)	—
Age group	—	—	—	—	*p* ^a^ = 0.095
Up to 29	4 (26.7)	6 (40.0)	5 (33.3)	15 (100.0)	—
30 or older	21 (35)	35 (58.3)	4 (6.7)	60 (100.0)	—
Gender	—	—	—	—	*p* ^a^ = 0.893
Male	14 (31.8)	25 (56.8)	5 (11.4)	44 (100.0)	—
Female	11 (35.5)	16 (51.6)	4 (12.9)	31 (100.0)	—
Tooth group	—	—	—	—	*p* ^b^ = 0.463
Incisors	8 (26.7)	16 (53.3)	6 (20.0)	30 (100.0)	—
Canines	6 (37.5)	8 (50.0)	2 (12.5)	16 (100.0)	—
Premolars	4 (50.0)	3 (37.5)	1 (12.5)	8 (100.0)	—
Molars	7 (33.3)	14 (66.7)	—	21 (100.0)	—
Lesion height	—	—	—	—	*p* ^b^ = 0.531
Supracrestal	5 (23.8)	15 (71.4)	1 (4.8)	21 (100.0)	—
Subcrestral	10 (32.3)	20 (64.5)	1 (3.2)	31 (100.0)	—
Extending to the middle third	9 (50.0)	4 (22.2)	5 (27.8)	18 (100.0)	—
Extending to the apical third	1 (20.0)	2 (40.0)	2 (40.0)	5 (100.0)	—
Circumferential distribution of the lesion	—	—	—	—	*p* ^b^ = 0.413
<90	6 (31.6)	12 (63.2)	1 (5.3)	19 (100.0)	—
90–180	12 (36.4)	20 (60.6)	1 (3.0)	33 (100.0)	—
181–270	2 (25.0)	4 (50.0)	2 (25.0)	8 (100.0)	—
>270	5 (33.3)	5 (33.3)	5 (33.3)	15 (100.0)	—
Proximity of the lesion to the root canal	—	—	—	—	*p* ^a^ = 0.793
Within dentin	14 (29.2)	30 (62.5)	4 (8.3)	48 (100.0)	—
With pulpal involvement	11 (40.7)	11 (40.7)	5 (18.5)	27 (100.0)	—

^a^Mann–Whitney test.

^b^Kruskal–Wallis test.

**Table 7:** Evaluation of ABL classification according to age group, gender, tooth group, lesion height, circumferential distribution, and proximity to the root canal.

Variable	Classification of alveolar bone loss			Total	*p*‐Value
	Mild	Moderate	Severe		
	*n* (%)	*n* (%)	*n* (%)	*n* (%)	
Total group	12 (16.0)	18 (24.0)	45 (60.0)	75 (100.0)	—
Age group	—	—	—	—	*p* ^a^ = 0.022 ^∗^
Up to 29	4 (26.7)	6 (40.0)	5 (33.3)	15 (100.0)	—
30 or older	8 (13.3)	12 (20.0)	40 (66.7)	60 (100.0)	—
Gender	—	—	—	—	*p* ^a^ = 0.610
Male	7 (15.9)	12 (27.3)	25 (56.8)	44 (100.0)	—
Female	5 (16.1)	6 (19.4)	20 (64.5)	31 (100.0)	—
Tooth group	—	—	—	—	*p* ^b^ = 0.388
Incisors	7 (23.3)	6 (20.0)	17 (56.7)	30 (100.0)	—
Canines	3 (18.8)	5 (31.3)	8 (50.0)	16 (100.0)	—
Premolars	2 (25.0)	1 (12.5)	5 (62.5)	8 (100.0)	—
Molars	—	6 (28.6)	15 (71.4)	21 (100.0)	—
Lesion height	—	—	—	—	*p* ^b^ = 0.007 ^∗^
Supracrestal^a^	2 (9.5)	6 (28.6)	13 (61.9)	21 (100.0)	—
Subcrestral^a^	1 (3.2)	10 (32.3)	20 (64.5)	31 (100.0)	—
Extending to the middle third^a^	5 (27.8)	1 (5.6)	12 (66.7)	18 (100.0)	—
Extending to the apical third^a^	4 (80.0)	1 (20.0)	–	5 (100.0)	—
Circumferential distribution of the lesion	—	—	—	—	*p* ^b^ = 0.106
<90	2 (10.5)	7 (36.8)	10 (52.6)	19 (100.0)	—
90–180	2 (6.1)	7 (21.2)	24 (72.7)	33 (100.0)	—
181–270	3 (37.5)	2 (25.0)	3 (37.5)	8 (100.0)	—
>270	5 (33.3)	2 (13.3)	8 (53.3)	15 (100.0)	—
Proximity of the lesion to the root canal	—	—	—	—	*p* ^a^ = 0.758
Within dentin	8 (16.7)	12 (25.0)	28 (58.3)	48 (100.0)	—
Within pulpal involvement	4 (14.8)	6 (22.2)	17 (63.0)	27 (100.0)	—

*Note:* If all letters in parentheses are different, this indicates a significant difference between the corresponding results.

^a^Mann–Whitney test.

^b^Kruskal–Wallis test with Conover’s multiple comparisons.

^∗^Significant difference at the 5.0% level.

**Figure 11:**
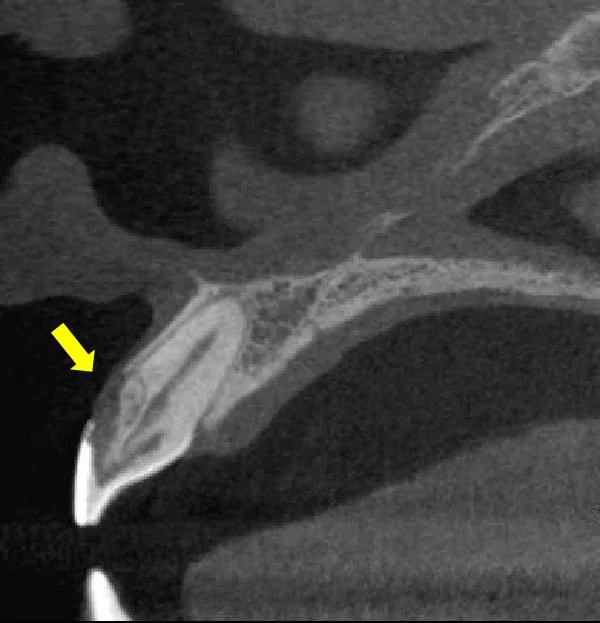
Illustrative image of the communication between subcrestal external cervical resorption and the periodontal ligament.

A significant association was found with ECR and the periodontal space lesion height (*p*  < 0.05), but not with circumferential distribution (*p*  > 0.05). Subcrestal ECR had the highest frequency. Twenty percent of lesions that reached the apical third communicated with the periodontal space. Lesions involving 90° to 180° were more likely to communicate (OR = 3.3), but this was not statistically significant (*p*  > 0.05). Lesion proximity to the root canal showed no significant association (*p*  > 0.05), despite a higher frequency when the pulp was involved (Table 8 and Figure 11).

**Table 8:** Evaluation of the communication between ECR and the periodontal condition according to age group, gender, tooth group, lesion height, circumferential distribution, and proximity to the root canal.

Variable	Communication of the resorption with the periodontal space		Total	*p*‐Value	OR (95% CI)
	Yes	No			
	*n* (%)	*n* (%)	*n* (%)		
Total group	52 (69.3)	23 (30.7)	75 (100.0)	—	—
Age group	—	—	—	*p* ^a^ = 0.369	—
Up to 29	12 (80.0)	3 (20.0)	15 (100.0)	—	2.0 (0.5–7.9)
30 or older	40 (66.7)	20 (33.3)	60 (100.0)	—	1.0
Gender	—	—	—	*p* ^b^ = 0.797	—
Male	30 (68.2)	14 (31.8)	44 (100.0)	—	1.0
Female	22 (71.0)	9 (29.0)	31 (100.0)	—	1.1 (0.4–3.1)
Tooth group	—	—	—	*p* ^a^ = 0.378	—
Incisors	18 (60.0)	12 (40.0)	30 (100.0)	—	1.0
Canines	12 (75.0)	4 (25.0)	16 (100.0)	—	2.0 (0.5–7.7)
Premolars	5 (62.5)	3 (37.5)	8 (100.0)	—	3.8 (0.9–15.8)
Molars	17 (81.0)	4 (19.0)	21 (100.0)	—	2.8 (0.8–10.5)
Lesion height	—	—	—	*p* ^a^ = 0.024 ^∗^	—
Supracrestal	13 (61.9)	8 (38.1)	21 (100.0)	—	^∗∗^
Subcrestral	26 (83.9)	5 (16.1)	31 (100.0)	—	^∗∗^
Extending to the middle third	12 (66.7)	6 (33.3)	18 (100.0)	—	^∗∗^
Extending to the apical third	1 (20.0)	4 (80.0)	5 (100.0)	—	^∗∗^
Circumferential distribution of the lesion	—	—	—	*p* ^a^ = 0.201	—
<90	11 (57.9)	8 (42.1)	19 (100.0)	—	1.00
90–180	27 (81.8)	6 (18.2)	33 (100.0)	—	3.3 (0.9–11.6)
181–270	5 (62.5)	3 (37.5)	8 (100.0)	—	1.2 (0.2–6.6)
>270	9 (60.0)	6 (40.0)	15 (100.0)	—	1.1 (0.3–4.3)
Proximity of the lesion to the root canal	—	—	—	*p* ^b^ = 0.234	—
Within dentin	31 (64.6)	17 (35.4)	48 (100.0)	—	1.0
With pulpal involvement	21 (77.8)	6 (22.2)	27 (100.0)	—	1.9 (0.7–5.7)

^a^Fisher’s exact test.

^b^Pearson’s chi‐square test.

^∗^Significant difference at the 5.0% level.

^∗∗^Not calculated due to very low frequency and consequently very wide intervals.

None of the variables included in the generalized linear mixed‐effects model showed a statistically significant association with the presence of ABL directly related to ECR (Table 9). The significant associations observed in the bivariate analysis for age group, tooth group, lesion height, and circumferential lesion distribution were no longer statistically significant in the generalized linear mixed‐effects model.

**Table 9:** Mixed‐effects logistic regression model for factors associated with alveolar bone loss directly related to external cervical resorption.

Variable	OR	95% CI	*p*‐Value
Age group	—	—	0.118
Up to 29	1.00	1.00	—
30 or older	3.03	0.71–20.77	—
Gender	—	—	0.819
Male	1.73	0.25–5.61	—
Female	1.00	1.00	—
Tooth group	—	—	0.213
Incisors/canines	1.00	1.00	—
Premolars/molars	3.09	0.50–21.92	—
Lesion height	—	—	0.639
Supracrestal	2.01	0.15–7.46	—
Subcrestal	2.98	0.33–19.65	—
Apical	1.00	1.00	—
Circumferential distribution of the lesion	—	—	0.520
≤180°	2.57	0.27–13.07	—
>180°	1.00	1.00	—
Proximity of the lesion to the root canal	—	—	0.601
With pulpal involvement	1.00	1.00	—
Within dentin	1.25	0.12–3.50	—

*Note:* Patient was included as a random intercept.

Abbreviations: CI, confidence interval; OR, odds ratio.

^∗^Significant difference at the 5.0% level.

## 4. Discussion

The hypothesis regarding the coronal‐apical extent and circumferential involvement of ECR was confirmed by the significant association between periodontal space lesion height and greater ABL in this cross‐sectional study. These findings indicate that more extensive ECR lesions are associated with greater adjacent ABL, supporting a relationship between lesion severity and periodontal involvement.

The sample comprised adults who underwent CBCT examination in a Brazilian clinical setting, with a predominance of males, and the findings were possibly associated with less frequent dental attendance and a higher incidence of dental trauma [18]. This pattern can also be observed in age distribution, particularly among middle‐aged individuals [6]. Factors such as dental trauma, previous orthodontic treatment, and periodontal disease tend to accumulate over time. This trend can, to some extent, explain the higher prevalence of ECR observed in this group.

CBCT enables quantification of the periodontal destructive potential of resorptive lesions through its high spatial resolution and multiplanar reconstruction capabilities. The use of a reduced FOV (50 mm × 50 mm) facilitated the detection of subcrestal and subgingival lesions, whereas previous studies employing larger acquisition fields reported lower detection of these characteristics, potentially underestimating early‐stage lesions [8, 10]. A previous Brazilian study using the same CBCT system but a longer acquisition time reported less detailed characterization of the dentogingival interface [10].

Consistent with these observations, the present analysis showed a predominance of lesions with subcrestal height and circumferential involvement ranging from 90° to 180°. These findings suggest that the quality of the DICOM dataset improved the identification of morphological features, which may partly explain the variability reported in previous studies [3, 14].

Involvement of the palatal/lingual surface was observed, contrasting with a previous ex vivo tomographic study that reported a predominance of the buccal surface, identified as the portal of entry [19]. This discrepancy may be related to differences in the tomographic device, FOV, and image resolution, particularly in radiographically challenging areas such as the palatal/lingual surface. However, concluding that the PreXion CBCT provides superior diagnostic performance would be premature.

Assessment of ABL patterns in cases of ECR showed a predominantly horizontal distribution, consistent with epidemiological findings in adult populations [9]. This pattern has been primarily attributed to periodontitis [9]. However, other authors suggested that local predisposing factors, such as dental trauma and orthodontic treatment, can also contribute to ECR development [5, 20]. Previous periodontal destruction may act as a risk marker as alterations in the periodontal microenvironment and inflammatory remodeling can predispose cervical root regions to resorption.

Considering the demographic profile of the study’s representative sample, a higher frequency of severe ABL was observed in teeth with resorptive lesions than in studies evaluating isolated bone loss, in which severity has been primarily associated with systemic factors, oral hygiene, and socioeconomic conditions [21]. In our study, the presence of resorptive lesions was associated with increased ABL, suggesting the involvement of an additional aggravating factor. This supports the multifactorial nature of periodontal breakdown.

Although accounting for multiple affected teeth within the same patient did not reveal significant associations with ABL in the mixed‐effects model, in contrast to some associations observed in the bivariate analysis, the wide CIs indicate considerable uncertainty in the estimates. This approach allowed a more detailed assessment of factors associated with ABL directly related to ECR, considering multiple lesion characteristics and different affected teeth within the same patient. To our knowledge, this aspect has received limited investigation in previous ECR studies [3, 6, 14]. Thus, these findings provide a basis for further investigation and characterization of the relationship between ECR and patterns of ABL.

The assessment of ABL in teeth with ECR revealed a predominance on the palatal/lingual surface. This study is the first to evaluate the spatial relationship between ECR and the distribution of ABL, whereas previous studies have primarily described the morphological characteristics of the resorptive lesion [3, 10, 15]. Including ABL as a periodontal parameter expands the understanding of the biological interaction between ECR and the periodontal supporting tissues, providing relevant information.

The predominance of lesions with subcrestal extent observed in the present sample was consistent with previous reports on ECR, which demonstrated a higher frequency of lesions in the middle third of the root [14, 22]. This finding suggests that, at the time of diagnosis of ECR, periodontal involvement may already be present, even in the absence of pulp involvement. Furthermore, angular measurements indicated that the crown–apical extent correlated more consistently with the severity of ABL than the circumferential distribution, which showed variable associations across tooth surfaces.

This variability supports the multifactorial nature of periodontal involvement and alveolar bone changes, which depend on local anatomical and functional factors, and suggests that two‐dimensional imaging modalities may underestimate lesion remodeling and periodontal involvement [18].

CBCT has been established as a reliable method for diagnosing and characterizing alveolar bone resorption, as it facilitates the assessment of circumferential lesion spread and the presence of multiple portals of entry, which are often undetectable on two‐dimensional Imaging [3, 18, 23]. In our study, communication with the periodontal width was significantly associated with the subcrestal height of the lesions, suggesting that periodontal involvement occurs at a more advanced stage. A despite this, CBCT demonstrated higher sensitivity and specificity for early detection and therapeutic decision‐making.

To the best of our knowledge, it has some limitations. Due to its retrospective design, access to detailed clinical information, including variables such as previous trauma and symptomatology. Although the large image records (*n* = 9208 patients) and the systematic assessment of three‐dimensional variables, including ABL, represent a novel approach in the literature, they enhance the robustness of the findings and partially mitigate this limitation.

Although CBCT provides high‐resolution three‐dimensional imaging, enabling early detection and accurate lesion characterization, it underestimates the extent of ABL at the microscopic level, particularly at the dynamic interface between resorption and repair. Nevertheless, it remains a reliable diagnostic tool compared with conventional imaging modalities.

Three‐dimensional analysis revealed an association between bone loss and cervical resorption, accounting for both vertical height and circumferential patterns. We observed an association between ECR characteristics and adjacent ABL, suggesting that periodontal involvement should be considered when evaluating the extent and potential clinical implications of cervical resorptive lesions. The subcrestal extension underscores the role of periodontal attachment in disease severity and prognosis. Future longitudinal studies are warranted to clarify the temporal progression of cervical resorption, determine whether pre‐existing ABL serves as a risk indicator, and support the development of radiological surveillance and early intervention strategies to clarify the practical implications of both conditions.

## 5. Conclusions

We can conclude as follows:

• The severity of ECR was associated with ABL, particularly in premolars and molars with increased subcrestal lesion height and wider circumferential extension.
• ABL related to ECR demonstrated a significant aggravating factor in multifactorial periodontal breakdown.
• Subcrestal extension into the periodontal tissues may contribute to ABL, even in early ECR in the absence of pulp involvement.

## Author Contributions

**Ana Paula de Medeiros Silva:** conceptualization, data curation, formal analysis, investigation, methodology, writing – original draft, writing – review and editing. **Thalles Gabriel Germano Lima**: conceptualization, formal analysis, investigation, methodology, writing – original draft, writing – review and editing. **Ana Beatriz Leitão de Albuquerque Wanderley**: conceptualization, investigation, methodology, writing – original draft, writing – review and editing. **Monikelly do Carmo Chagas Nascimento**: conceptualization, data curation, formal analysis, investigation, methodology, writing – review and editing. **Daniela Siqueira Lopes**: conceptualization, data curation, formal analysis, investigation, methodology, writing – original draft, writing – review and editing.

## Funding

This study did not receive any specific funding.

## Disclosure

All authors have read and approved the final version of the manuscript. The corresponding author had full access to all of the data in this study and takes complete responsibility for the integrity of the data and the accuracy of the data analysis.

## Conflicts of Interest

The authors declare no conflicts of interest.

## Supporting Information

Additional supporting information can be found online in the Supporting Information section.

## Supporting information


**Supporting Information** The checklist recommended by the Strengthening the Reporting of Observational Studies in Epidemiology (STROBE) checklist is provided as Supporting Information for this cross‐sectional study.

## Data Availability

The data that support the findings of this study are available from the corresponding author upon reasonable request.
